# Motoring Notes

**Published:** 1909-12-11

**Authors:** 


### Motoring Notes.

In my last notes allusion is made to the cars of a similar model and date belonging to two different owners, one of whom has never any trouble, whose engine always starts when wanted and runs as long as it is needed without any of the exasperating breakdowns on the road experienced by the other, who sooner or later comes to the conclusion that he has bought the worst specimen of engine ever built, while his more fortunate neighbour succeeded in getting the best.

Now, coming from the same builder at about the same time, there was no practical difference between the two motors at first; their construction was the same and their running qualities equally good at the outset, but one grows worse with each day's use and the other seems to get better, if anything, the more it is worked. The wide difference in the care given the motors is reflected by their condition, and a glance suffices to indicate it. The hammering and clatter with which one runs is in keeping with its slovenly grease and dirt-covered appearance, with every bearing and adjustment loose and shaking. The clean engine is a satisfaction to its user as well as to all who have occasion to see it. With all its adjustments carefully made, every bearing well oiled, it works sweetly and with such energy as to indicate that it is developing all the power at which it is rated.

But no engine will continue to perform in this manner unless the detailed work called for is properly attended to. Most of these details are insignificant in themselves, and the attention required altogether does not exceed fifteen or twenty minutes daily, if the car be out that frequently; if not, every time it is made use of. It is neglected that time may be saved in getting away; but what is apparently gained in the garage is more than lost through the necessity for increasingly frequent roadside adjustments, which, by the way, are usually unsatisfactory, and particularly when performed in the manner customary with those who make a habit of neglecting their engine. This is, sooner or later, followed by the time when the engine refuses any longer to respond, when its petty ailments have combined to assume a more serious state and, either together or alone, are more than sufficient to prevent its running. Then the fifteen minutes' inspection that should have been given every time the engine was used has grown to a period equal to that quarter-hour multiplied by the number of times it has not been devoted to inspection and adjustment, and the result is, as often as not, a total dismantling of the engine, and a bill for a day or two's time at a shilling or more an hour only partly represents the cost of this constant neglect. Exactly what this daily care should consist of will differ in detail with different makes of cars; but, as a general rule, instructions of this kind will apply equally well to any make, as the object in every instance will be the same—the prevention that is so much more effective than a deferred cure. Many nuts that should be pinned or otherwise locked are wanting in this essential, and are apt to work loose; every moving part of the engine is subject to the same failing unless properly looked after, while it should hardly be necessary to caution the owner of average common sense that excess oil and grease are mud and dust attractors, and should not be allowed to remain. Faulty adjustment, that means but a turn or two of the spanner, if taken when first discovered, may mean a new bearing if allowed to go on unheeded. Take up the main bearings whenever required, watch those of the connecting rods, the gears, the chains, especially the latter, for faulty alignment here means new sprockets sooner or later. There are a hundred things of this kind that will suggest themselves to the careful owner, and most of them are mentioned in the manufacturers' instructions. They are the little things that count, and neglect of them means eternal dissatisfaction and heavy repair bills. The most fool-proof machine ever constructed, nay, one even built on heavenly lines, would not long satisfy the man who persistently neglects it and, in doing so, violates every law of mechanics, as well as the dictates of common sense.

Some Practical Notes.

If the springs of a car have become very much rusted, the only cure is to take them down. This will necessitate jacking up the frame, not the axle, and supporting it while the spring shackles and clips are released. The springs will then have to be dismounted, each individual leaf cleaned with emery-cloth, well lubricated with grease, and remounted. One spring should be done at a time, so that the leaves, bolts, etc., may not become mixed.

Leaky tyre-valves are often a very great source of annoyance to motorists. By jacking up the car and turning the wheel so that the valve is at the extreme top, it is an easy matter by the aid of a glass of water to see if the valve is airtight. Sometimes the leak occurs at the foot of the valve; this is often caused by the valve-tube having too large a hole punched through it, and consequently the gripping power of the bridge-piece is lost. The only remedy for this is a new valve-tab.

Motorists of experience know that the high-tension current is very difficult to insulate and always ready to leak away through the most unlikely channels. If the ignition acts strangely and the trouble cannot be located, it is advisable to look for a high-tension leak. The elusive current will frequently follow oily wood or cloth, if given an opportunity, causing the most mysterious short circuits.

